# Screening for Congenital Cytomegalovirus Infection in Newborns

**DOI:** 10.3390/v18010063

**Published:** 2025-12-31

**Authors:** Junfeng Zhang, Jiajia Cao, Qing Ye

**Affiliations:** Department of Clinical Laboratory, The Children’s Hospital, Zhejiang University School of Medicine, National Clinical Research Center for Child Health, National Children’s Regional Medical Center, Hangzhou 310052, China; chzjf@zju.edu.cn (J.Z.); jiajiacao@zju.edu.cn (J.C.)

**Keywords:** cytomegalovirus, congenital infection, newborns, CMV

## Abstract

Congenital cytomegalovirus infection is an underrecognized congenital infection. Globally, it impacts approximately 1 of every 200 live births. Although infected infants can have an increased risk of long-term sequelae, such as neurodevelopmental impairments and sensorineural hearing loss, most of the infected infants do not show visible signs at birth. As congenital cytomegalovirus infection often goes undetected and screening programs are not widely accepted, awareness of congenital cytomegalovirus in neonates is lacking. The aim of this study is to offer the current status of the epidemiology, clinical manifestations, and laboratory testing for the diagnosis of congenital cytomegalovirus infection and newborn screening approaches.

## 1. Introduction

Congenital cytomegalovirus (cCMV) infection ranks as the most common congenital viral infection among newborns globally. The reported overall prevalence of cCMV infection in newborns is approximately 0.64% [1]. It is a leading non-genetic factor contributing to neonatal sensorineural hearing loss (SNHL) and a significant cause of neurodevelopmental delay [1,2,3]. Every year in the United States, approximately 40,000 infants are born with cCMV infections, causing an estimated 400 deaths, and 8000 develop permanent neurological complications [4,5]. The cost of cCMV is estimated at USD1–USD2 billion per year [6,7]. The burden of disease is tremendous because the incidence of cCMV infection is notably greater than that of several disorders included in current newborn screening programs [8], and cCMV-related disabilities among newborns are estimated to be more common than other well-known diseases, like Down syndrome [9]. Neonates with symptomatic infection refers to the presence of visible signs at birth suggestive of congenital infection, while asymptomatic refers to being without any obvious clinical abnormalities [10]. Neonates affected by cCMV can present a diverse range of signs, develop long-term sequelae, and in some cases, even face death [3,10]. Nearly 90% of cCMV-infected newborns do not present any obvious clinical abnormalities at birth [11,12]; if symptoms are present, they are frequently nonspecific [9,13]. Therefore, most newborn infections are not recognized at birth. In addition, sequelae that occur during cCMV infection often have a delayed onset. Considering the evidence regarding the effectiveness of early intervention for children with SNHL and the availability of antiviral treatment [14], screening strategies to identify cCMV infection as early as possible are needed. Herein, we addressed the epidemiology and diagnostic methods of cCMV infection, as well as screening for neonatal cCMV.

## 2. Epidemiology

**Figure 1:**
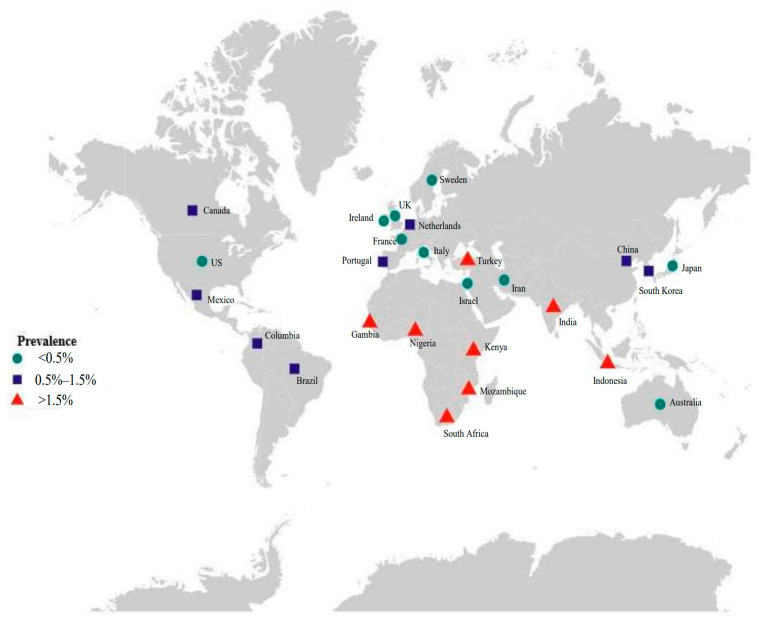
Birth prevalence of congenital CMV infection.

Cytomegalovirus (CMV) is a common infection that infects a majority of people worldwide, and approximately 83% of the general population is seropositive [15]. Several studies have estimated that the seroprevalence among women of childbearing age ranges from 70% to 92% across different regions, and the global rate is 86% [15,16]. CMV can be transmitted from mother to fetus; therefore, cCMV is the most common form of intrauterine infection [17]. cCMV infection in high-income countries occurs in approximately 0.48% of all live births, while it occurs in approximately 1.42% of all live births in low- to middle-income countries [18] and is more common in South Africa and Indonesia (Figure 1). When focusing on cCMV in newborns, the status of CMV in pregnancy should also be known.

**Figure 2:**
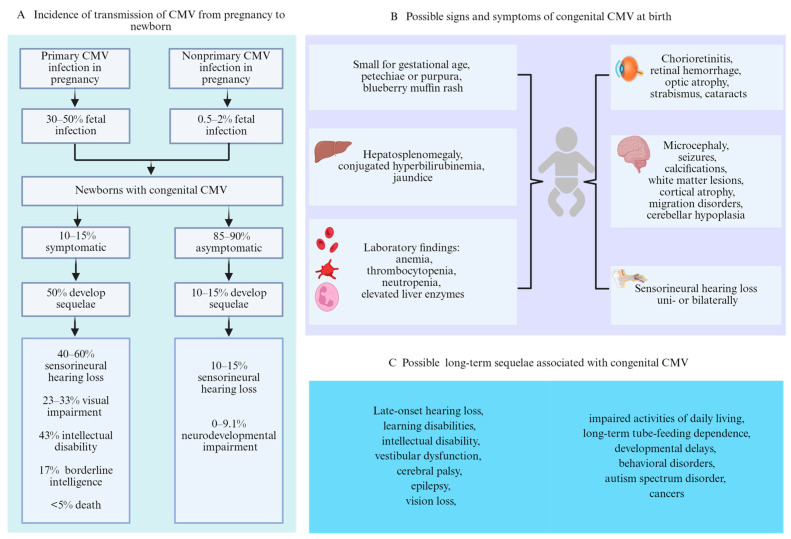
Transmission, possible signs and symptoms at birth, and long-term sequelae of congenital CMV. (**A**) Incidence of transmission of CMV from pregnancy to newborn. (**B**) Possible signs and symptoms of congenital CMV at birth. (**C**) Possible long-term sequelae associated with congenital CMV.

The first infection with CMV in pregnancy is referred to as primary infection, and the incidence is approximately 1% to 2%, whereas the reactivation of latent virus or exposure to a different strain, referred to as nonprimary infection, is reportedly as high as 10% of pregnancies [19,20]. The risk of fetal transmission is contingent upon the kind of maternal infection. Primary infection is associated with a 30–50% risk of fetal transmission [10,21,22], whereas nonprimary infection is associated with a lower risk (0.5% to 2%) [22,23] (Figure 2A). Previous studies have demonstrated that 10–15% of neonates are born with visible signs or central nervous system (CNS) involvement at birth, whereas 85% to 90% of neonates will be born with no symptoms [13,24,25]. Approximately 50% of newborns with symptoms at birth are at risk of developing long-term sequelae [26]. Among those who are asymptomatic at birth, 10–15% may develop sequelae such as SNHL [27,28] (Figure 2A).

Although CMV transmission from mother to infant is more likely in women with primary infection at the population level, the majority of symptomatic newborns with cCMV are born from mothers with nonprimary infection. Maltezou et al. found no statistically significant differences in the incidence of symptomatic cCMV infection at birth or the development of long-term sequelae between primary and nonprimary maternal infections [29]. This study differs from widely recognized previous studies. This may be related to the different definitions of maternal primary and nonprimary CMV infection during pregnancy, and the eligible studies did not provide complete data in the meta-analysis; thus, further data collection is needed. Once the fetus is congenitally infected, the risk of long-term sequelae is similar in children born post-primary and nonprimary maternal CMV infection. The need to focus interest on maternal nonprimary infection is supported by many experts worldwide.

## 3. Clinical Manifestations

Neonates affected by cCMV can present a diverse range of symptoms, signs, and long-term sequelae (Figure 2B,C), although the majority have no recognizable apparent signs at birth [14]. Newborns with cCMV infection present clinical abnormalities at birth, which are commonly referred to as symptomatic cCMV infection, and can affect multiple organs, especially the CNS and reticuloendothelial system. In general, a broad range of clinical features of cCMV infection among those symptomatic at birth include any of the following: jaundice, hepatosplenomegaly, petechial rash, blueberry muffin rash, chorioretinitis, pneumonia, or small for gestational age [14]. CNS involvement manifests as microcephaly, intracranial abnormalities (ventriculomegaly, cortical malformations, periventricular calcifications, and/or germinal cysts), seizures, chorioretinitis, and SNHL [30,31]. For infants with CMV-related clinical signs, the most common finding is petechial rash, followed by thrombocytopenia and microcephaly [26,31].

The laboratory findings were abnormal, and more than half of the newborns with symptomatic infection had hyperbilirubinemia, thrombocytopenia, and elevated liver transaminase levels [31]. Thrombocytopenia hits its lowest point during the second week after birth and returns to the normal level within 3 to 5 weeks after birth, whereas the levels of bilirubin and transaminase reach their peak within the initial 2 weeks of life and may continue to rise for several weeks [11]. The definition of symptomatic congenital CMV disease is different. In a report, the International Congenital Cytomegalovirus Recommendations Group has made efforts to provide recommendations for standardized definitions of cCMV infection and disease. Mild is defined as one or two isolated, mild, and transient manifestations, while moderate to severe is defined as multiple manifestations and/or CNS involvement [14]. The older literature reflects that the presence of SNHL alone at birth is still considered asymptomatic. Recently, according to a European congenital cytomegalovirus initiative, physical examination, laboratory results, neuroimaging, hearing, and ophthalmologic evaluation are required to classify the infection as symptomatic or asymptomatic and it is important to note that isolated SNHL is included in the symptomatic onset [32].

However, regardless of whether clinical symptoms occur at birth, some neonates with cCMV infection have long-term sequelae during follow-up, mainly including progressive visual impairment, hearing impairment, and mental retardation or less severe cognitive impairment [10,33]. Among infants with symptomatic cCMV, between 23% and 33% will develop visual impairment [10,34], 43% will have intellectual disability, 17% will have borderline intelligence [26], and the mortality rate is estimated to be less than 5% [4] (Figure 2A). According to a large population study, the prevalence of moderate to severe sequelae was 32%, and these sequelae were identified in the first year of life in contrast to the mild impairment identified at 2–7 years [12]. SNHL, which is the most common late-onset sequelae of cCMV, occurs in 10 to 15% of asymptomatic infants and 40% to 60% of symptomatic infants [28,35]. In a report by Salomè et al. [36], none of the asymptomatic infants developed a stable SNHL, while 14% showed a variable hearing impairment. The proportion of infants with a single clinical manifestation at birth who develop SNHL is significantly lower than that with multiple clinical manifestations (31% vs. 53%) [31]. Symptomatic cCMV in infants increases the likelihood of bilateral and severe/profound hearing loss; 30–40% will experience SNHL during the neonatal period or in the first years of life, 20–54% may experience progressive hearing loss, and 18–27% may develop delayed-onset hearing loss at a median age of 33 months [37,38]. Asymptomatic neonates may experience SNHL during the first several years of life, 38% of whom develop delayed-onset hearing loss at a median age of 44 months and are more likely to have unilateral and fluctuating hearing loss [37,38]. In addition, the incidence of neurodevelopmental impairment in children with asymptomatic cCMV has been reported to range from 0% to 9.1% [27].

## 4. Laboratory Testing

**Figure 3:**
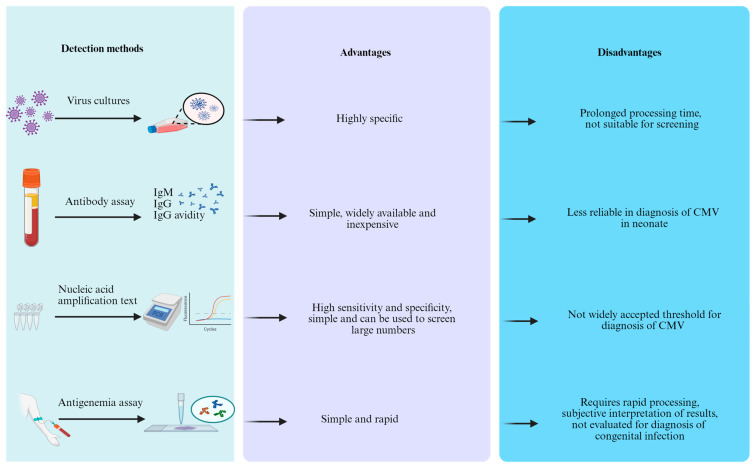
The detection methods, advantages, and disadvantages of congenital CMV in newborns.

Laboratory testing is often a necessary approach to identify congenital CMV infection (Figure 3).

### 4.1. Virus Isolation Methods

CMV detected by virus isolation was considered the standard method prior to the introduction of the nucleic acid amplification test. Urine or saliva is generally inoculated into human embryonic fibroblasts for 2–4 weeks or longer, which are then detected via light microscopy to observe the appearance of the characteristic cytopathic effect of CMV. The excretion of CMV was not detected in newborns, and over 60% of healthy infants by 5 months of age experienced primary infection without clinical manifestations, and that virus excretion diminishes at 10–12 months of age [39]. Thus, virus isolation occurs in the initial two weeks after birth; otherwise, subsequent virus shedding might be caused by neonatal infection or postnatal exposure to breastmilk or blood products that acquire CMV [40]. Over the past few decades, with the development of rapid culture methods, the results have shortened to 24–36 h [41,42]. A microtiter plate fluorescent antibody assay with 94.5% sensitivity and 100% specificity was used to detect CMV in urine samples for rapid detection [43], and this microtiter plate method for detecting CMV in the saliva of newborns with congenital CMV infection was also evaluated [44]. Although growth of the virus has excellent specificity, it has low sensitivity, complex procedures, is time-consuming, and is not suitable for widespread screening purposes. It has been supplanted by molecular methods.

### 4.2. Antibody Assay

Antibody detection for CMV infection mainly includes IgM, IgG, and IgG avidity antibodies [14]. In the past, several assays, including indirect hemagglutination, complement fixation, anticomplement immunofluorescence, and radioimmunoassay, have been used to detect IgM antibodies [45]. In addition, enzyme-linked immunosorbent assay (ELISA) has been more widely used, and IgM capture ELISA employing labeled F(ab′)_2_ fragments could avoid false-positive results [40,46]. Sensitivities are 54% to 100%, and specificities range from 62% to 100% in different CMV IgM assays [19,47,48,49,50,51,52,53]. IgM antibodies are produced for the first time within 1–2 weeks after primary infection and can persist for a long time. Generally, the levels of IgM antibodies decrease to undetectable levels within 4 months, but in a minority of individuals, IgM antibodies can remain positive for up to 9 months to 1 year. On the other hand, IgM antibodies can also be detected when viruses reactivate [54]. Thus, distinguishing acute primary infection from recurrent infection is difficult. Additionally, it has questionable specificity and sensitivity for the diagnosis of cCMV. In particular, IgM antibodies may cross-react with other viral infections. For instance, the Epstein–Barr virus can lead to false-positive results [55]. Therefore, only relying on the negativity or positivity of IgM antibodies is not sufficient to diagnose primary CMV infection.

IgG-specific antibodies appear 1–2 weeks after CMV infection, peak 4–8 weeks later, and can persist for several years or even for life. The overall estimate of CMV IgG seroprevalence was 41.9% in developed countries [16]. In infants under 1 year of age, the seroprevalence was 24.7% in females and 21.9% in males [56]. Clearly, seroconversion of IgG antibody changes from negative before pregnancy to positive is considered a diagnostic indicator of primary maternal infection, and the determination of IgG shows 97% to 100% sensitivity and 97% to 100% specificity in commercial CMV IgG testing [19]. However, for neonates, a positive result may be due to maternal CMV IgG antibodies crossing the placenta. Most investigators suggest that the detection of CMV IgG is not useful for the diagnosis of previous exposure to CMV [57]; however, a negative result reduces the probability of cCMV [10]. The detection of CMV IgG avidity is a critical assay that has a significant diagnostic value for primary infections and is related to the risk of transmission to the fetus [58,59,60]. With the majority of assays, low avidity indices (less than 30%) indicate a recent CMV primary infection within the last 3–4 months, whereas high avidity indices (greater than 60%) are highly suggestive of a nonprimary infection [57,61]. Vilibic-Cavlek et al. [62] successfully used the CMV IgG avidity test to diagnose cCMV in newborns and infants. In this study, 40 infants under 1 year of age with suspected cCMV infection were evaluated; 13 were positive for CMV IgM antibodies, and 3 had equivocal IgM results. In terms of IgG avidity, 8 out of 13 (61.5%) had low avidity indices in the group with positive IgM antibodies, and 13 out of 24 (54.2%) with negative IgM antibodies had low avidity indices. In terms of age distribution, the study demonstrated that IgG avidity is valuable for the diagnosis of CMV infection regardless of the IgM result among children older than 3 months of age. Maternal IgG antibodies derived from the placenta have a high avidity index, which affects the IgG avidity of infants younger than 3 months, and CMV infection should be further confirmed by other methods, such as molecular assays.

### 4.3. Antigenemia Assay

The use of pp65-specific monoclonal antibodies to detect CMV antigens in polymorphonuclear leukocytes can help to diagnose CMV infection and monitor treatment in immunocompromised patients [63]. Quantitative results were obtained by counting and reporting the number of positive cells among 2 × 10^5^ peripheral blood leukocytes [64]. Moreover, the evaluation of antigen-positive cells significantly correlates with the severity of CMV infection [65]. Studies have shown that the pp65 antigenemia assay yields comparable results to techniques based on molecular amplification [66]. This assay must be processed immediately after sample collection. Moreover, there are issues such as subjectivity in quantification and lack of standardization across different laboratories [63]. Another limitation is that it requires an adequate number of neutrophils, which may not be possible in newborns because of difficulty in obtaining sufficient blood. Furthermore, this method has been less commonly evaluated for the diagnosis of cCMV infection in newborns.

### 4.4. Nucleic Acid Amplification Test

Polymerase chain reaction (PCR) for detecting CMV in the urine or saliva should be carried out within the initial 3 weeks after birth and ideally before 14 days [13,67]. Studies have shown that the sensitivity and specificity of PCR are high for both saliva and urine samples in screening for cCMV-infected newborns [68,69,70,71]. Infected newborns shed large quantities of virus in the urine, and the PCR methods used for detecting cCMV in the urine have 100% sensitivity and 99% specificity [67]. It is recommended that within 21 days of birth, a negative result of a urine sample by PCR in a neonate is considered sufficient to exclude cCMV infection and is not necessary for repeat sampling [13].

Recently, the Simplexa congenital CMV direct real-time PCR assay, which requires approximately 60 min, has been applied to both neonatal urine and saliva swab samples. Fernholz EC et al. validated that the clinical sensitivity of saliva swabs and urine was 100% and 91.18%, respectively, and the clinical specificity reached 100% for urine and 96% for saliva swabs [72]. The findings indicated that, when compared with laboratory-developed PCR, this assay demonstrated excellent performance and led to a faster implementation timeline. However, collecting urine from a neonate may be difficult, and some urine collection methods, such as the use of cotton balls in diapers [73] and filter paper [74] analyzed by PCR for the detection of cCMV infection, are considered feasible.

Saliva samples are obtained from a cheek swab, and according to different studies, the sensitivity of saliva PCR ranges from 97.4% to 100%, and the specificity ranges from 91.5% to 99.9% [69,75]. The false-positive rates varied between 7% and 48% [69,76,77,78]. In a large study involving 34,989 newborns at birth, real-time PCR assays conducted on dried and liquid saliva demonstrated high sensitivity (97.4–100%) and specificity approaching nearly 100% when compared with rapid saliva culture for the detection of cCMV infection [69]. While saliva is easier to obtain than urine, a positive CMV test could yield false-positive PCR results thought to be due to human milk contamination through breast- or bottle feeding [79,80]. However, in a study by Ross S.A. et al., 74,788 infants were screened at birth, of which 307 infants tested positive for CMV through saliva PCR, with 284 confirmed to be CMV-positive. They concluded that 7.5% (23 out of 307) false-positive rates suggest that the results of saliva PCR are not likely to be significantly affected by breastfeeding or other perinatal exposures [78].

The diagnostic efficacy of saliva and urine samples for congenital infections in newborns has been controversial. A study evaluated the usefulness of saliva and urine samples collected from newborns for the screening of cCMV infection. The fact that there was a 99.7% agreement between the PCR results of the saliva and urine samples indicated that saliva samples are equally reliable as urine samples when it comes to detecting cCMV infection [81]. However, Gunkel J et al. demonstrated that the sensitivity of saliva is lower than that of urine for the detection of postnatal CMV infection in preterm infants [82]. Other studies have shown that the specificity of saliva PCR is lower than that of urine PCR [75,77,80]. Moreover, false positives of saliva PCR may be further reduced. This can be achieved by collecting the sample prior to feeding or at least one hour after breastmilk intake. Additionally, a positive saliva PCR result ought to be verified by testing either a second saliva sample or a urine sample [13,79].

A higher viral load in the urine or blood at birth is closely related to symptomatic disease in newborns [83] and the risk of sequelae. Previous reports demonstrated that a high CMV viral load was linked to an elevated risk of SNHL in the neonatal or later period [84,85,86] and with neurological sequelae [87]. A 6-year follow-up study revealed that asymptomatic neonates with cCMV infection and viral loads in blood greater than 12,000 copies/mL had a higher probability of developing late-onset disease sequelae and that a viral load greater than 17,000 copies/mL increased the risk of hearing deficit [88]. Conversely, Kabani N et al. reported that although symptomatic infants with SNHL have higher viral loads in both urine and saliva than those with normal hearing at birth, viral loads are similar in asymptomatic infants with and without hearing loss [89]. These results suggest that the viral load is not useful for the prediction of hearing loss during cCMV infection. A positive CMV PCR result of cerebrospinal fluid in newborns may be considered a CNS injury in cCMV infection; however, the extremely low positive prevalence rate of 13.7% suggests that its practical significance in clinical settings is rather limited [90]. In summary, there is currently no standardized level or cutoff value for detecting saliva, urine, blood, or other sample types in newborns, and there is no absolute correlation between the viral load and the risk of cCMV infection development or deterioration.

### 4.5. Dried Blood Spot Testing

Dried blood spot (DBS) samples were routinely collected from all newborns for metabolic screening in many countries. In the past few decades, PCR assays of neonatal DBS have been retrospectively used for the detection and diagnosis of cCMV in infants over 3 weeks of life [91,92,93,94,95]. In a study of 20,448 infants, DBS PCR assays were performed for newborn CMV screening. It demonstrated that when compared with rapid saliva culture, the CMV testing using DBS real-time PCR showed a relatively low sensitivity, which was merely 34.4% [96]. The low sensitivity could be due to the methodology used for DNA extraction or the testing methods because the method employed for extraction from DBS is a major factor in analytic sensitivity [97]. Recently, there has been an improvement in the sensitivity of the assay. In 2021, a prospective screening study involving 12,554 newborns by Dollard et al. demonstrated relatively high analytical sensitivity (73.2% and 76.8% from two laboratories) for DBS and the combined sensitivity of both laboratories reached 85.7% [76]. The increased sensitivity may be related to improvements in the extraction method and DBS elution buffer. Furthermore, droplet digital PCR was assessed when carried out directly on the DBS punch without nucleic acid extraction. This approach aimed to significantly reduce the sample size and enhance the feasibility of newborn screening for cCMV using DBS samples [98]. However, the detection of CMV varied or was not possible by all methods. This might be attributed to the extremely low viral loads present in DBS; therefore, a negative result of DBS PCR cannot definitively rule out cCMV [10,13,98].

## 5. Screening

At present, seroconversion of IgG antibody is identified for determining primary maternal infection. However, preconceptional serologic screening for CMV is not carried out routinely in many countries [14], so seroconversion data may not always be accessible. As a surrogate method, the detection of CMV IgM and low-avidity IgG is useful for the serologic diagnosis of primary infections [99]. Screening might be useful because treatments with antivirals such as valacyclovir have been reported to show a reduction in the risk of transplacental transmission following maternal primary CMV infection in the first trimester [100]. CMV serological screening in the first trimester, followed by oral valaciclovir treatment, shows cost-effectiveness [101].

**Figure 4:**
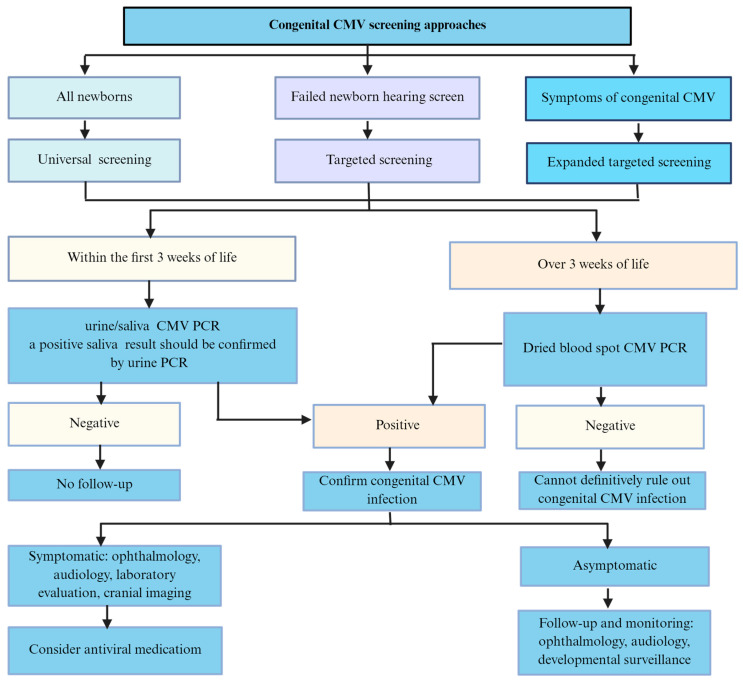
Screening approaches for congenital CMV in newborns.

The screening for congenital CMV infection in neonates includes universal CMV screenings of all infants and targeted CMV screenings of newborns who fail the hearing screening (Figure 4). Universal CMV screening involves testing urine, saliva, and/or DBS on all newborns. Both urine and saliva samples from newborns are useful and reliable for CMV screening because of their high sensitivity and specificity [69,81]. DBS is an easily accessible sample for universal screening, as it is collected from all infants at birth in many countries.

At present, universal screening for cCMV in all newborns does not routinely occur in most countries [23]. The majority of infants with cCMV are asymptomatic at birth, making it challenging to identify cCMV infection without universal newborn CMV screening. Universal newborn CMV screening provides an important opportunity to identify asymptomatic cCMV infants who do not exhibit clinical features at birth but have an elevated risk of developing late-onset SNHL [14], leading to timely diagnosis and a comprehensive assessment of all infants with cCMV. Therefore, infants with cCMV could benefit from universal CMV screening. Targeted cCMV screening could be limited to those newborns whose hearing screening fails and who can then be directed to undergo evaluations to determine whether congenital infection exists. Currently, the practice of targeted cCMV screening is becoming more prevalent in the United States. In a retrospective study, Stehel EK et al. performed a targeted CMV screening approach and reported that 6% of newborns with confirmed hearing impairment were attributable to cCMV infection [102]. Another study showed that DBS PCR indicated cCMV infection was the cause in 10% of infants who were diagnosed with SNHL within the first two months of their lives [103]. Moreover, a large multicenter study revealed that 57% of newborns with CMV-related SNHL were identified via a targeted CMV screening approach [104]. Targeted CMV screening would benefit fewer children, and many hospitals have suggested that targeted CMV screening may be offered for newborns who have no clear responses on hearing screens for identifying infants with CMV-related SNHL at birth [28,105].

Additionally, expanded targeted screening is another approach. In this approach, infants with clinical manifestations, such as intrauterine growth restriction, small for gestational age, thrombocytopenia/petechiae, hyperbilirubinemia, hepatosplenomegaly, hepatitis, microcephaly, and abnormal findings on cranial ultrasound or MRI, trigger clinicians to test newborns for cCMV infection [106,107]. Although cases of asymptomatic cCMV infection or exhibiting inapparent clinical manifestations only at birth may be overlooked, it is beneficial for diagnosing symptomatic cCMV infection in newborns until universal screening is introduced [108]. Data from prospective cohorts have shown that both targeted and universal newborn cCMV screening seem to be cost-effective [109]. A recent study reported that compared with targeted cCMV screening, universal cCMV screening could prevent more cases of severe hearing loss [110]. The value of providing early intervention for children with cCMV could improve outcomes [111]. Some experts have suggested that universal screening should be taken into consideration because it is beneficial for the early detection of cCMV infection and intervention for SNHL, where appropriate [14]. Targeted screening for cCMV in infants who did not pass the hearing test is especially useful. These results suggest that it is warranted to carry out newborn cCMV screening.

## 6. Conclusions and Future Directions

cCMV infection has a significant impact worldwide because of its widespread prevalence in infants and the long-term burden of the potential severity of its sequelae. It is difficult to diagnose cCMV infection in the first month of life, as most neonates infected with cCMV do not show obvious signs of infection. The progress of laboratory diagnostics has helped to identify newborns affected by cCMV quickly and accurately. The newborn CMV screening approach is critical for early detection and interventions, and the practice of screening appears to be positive.

## Data Availability

No data were used for the research described in the article.

## Abbreviations

The following abbreviations are used in this manuscript:

cCMV	Congenital cytomegalovirus
CMV	Cytomegalovirus
SNHL	Sensorineural hearing loss
CNS	Central nervous system
ELISA	Enzyme-linked immunosorbent assay
PCR	Polymerase chain reaction
DBS	Dried blood spot
